# Supplementary material for: Fusion dynamics of cubosome nanocarriers with model cell membranes
Source: Nat Commun. 2019 Oct 3;10:4492. doi: 10.1038/s41467-019-12508-8 (PMC6776645; doi:10.1038/s41467-019-12508-8)
Supplement: Supplementary file 1 — Supplementary Information [file 41467_2019_12508_MOESM1_ESM.pdf]

Supplementary Information

Fusion dynamics of cubosome nanocarriers with model cell membranes

*Brendan P. Dyett[#], Haitao Yu[#], Jamie Strachan, Calum J. Drummond and Charlotte E. Conn**

School of Science, College of Science, Engineering and Health, RMIT University, Victoria, Australia

*** Corresponding author:**

A/ Prof. Charlotte E. Conn

Phone: +61 3 9925 4265

Email: charlotte.conn@rmit.edu.au

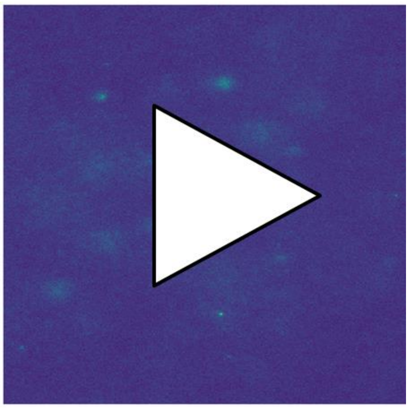

Supplementary Video 1. Interaction of MO cubosomes with DOPC bilayer. Available online. 4x playback speed

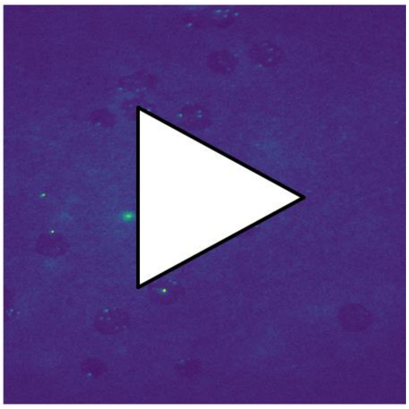

Supplementary Video 2. Interaction of 1% DOTAP cubosomes with DOPS/DOPC bilayer. Available online. 4x playback speed.

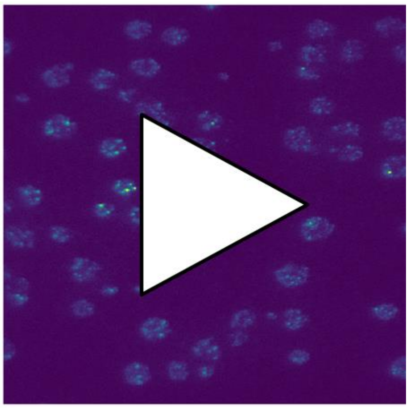

Supplementary Video 3. Interaction of 1% DOTAP cubosomes with DOPS/DOPC bilayer in buffer environment. Available online.4x playback speed.

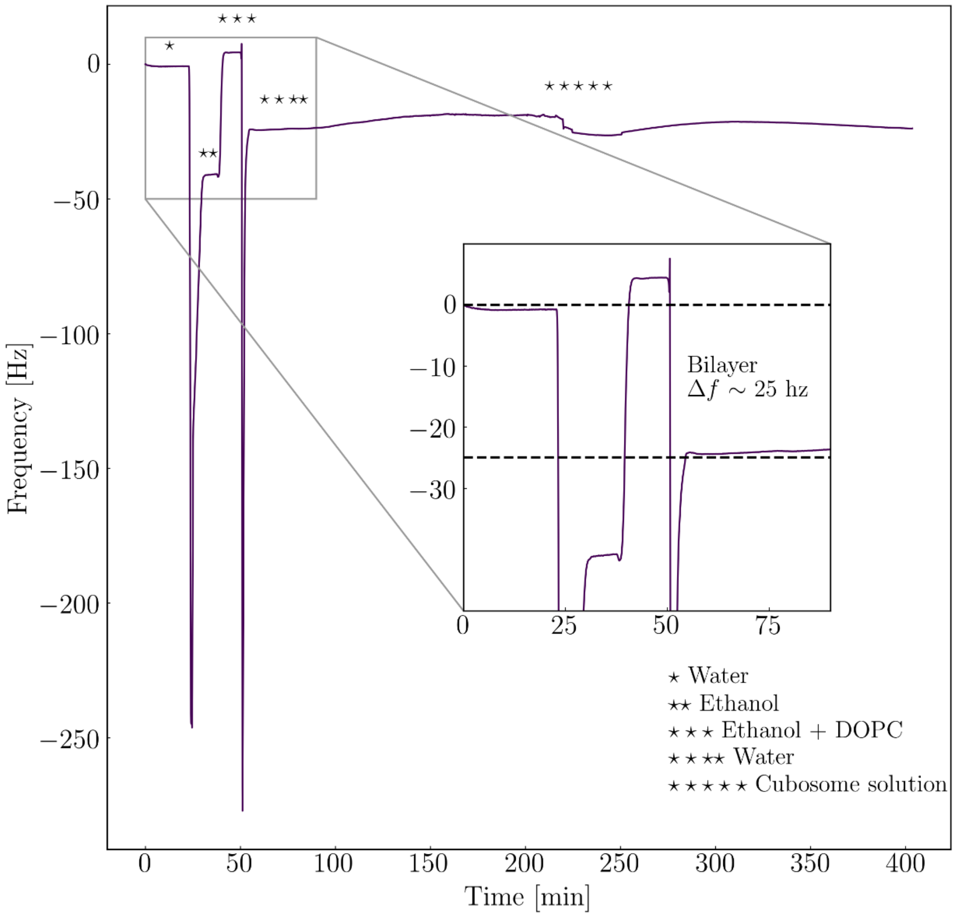

Supplementary Figure 1. Frequency shift from QCM-d measurements during the formation of a DOPC bilayer by solvent exchange. The stages of formation are highlighted by * indicating the solution being introduced to the fluid cell. The inset highlights the stage of bilayer formation as the solution transfers from water – to ethanol – to ethanol with DOPC – to water. The transfer from the starting water environment and final water environment demonstrates a frequency shift of ~ 25 Hz corresponding to the formation of a complete single bilayer.

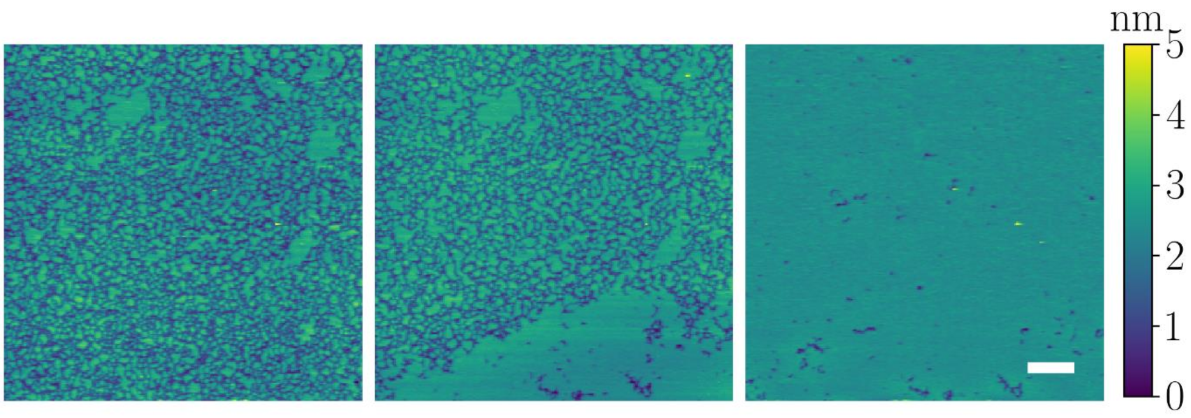

Supplementary Figure 2. AFM height image showing the in-situ formation of a DOPC bilayer by solvent exchange. Scale bar = 500 nm.

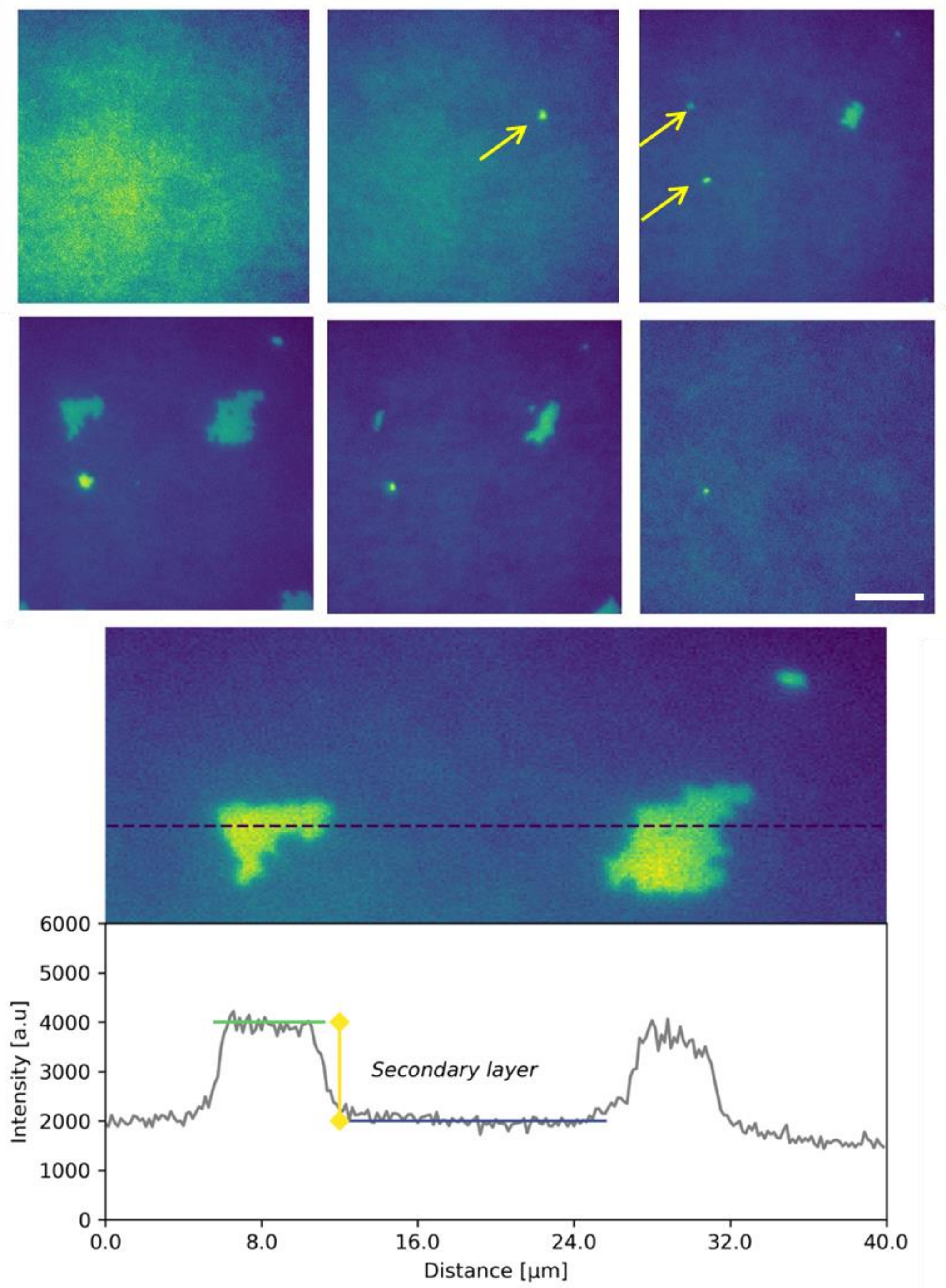

Supplementary Figure 3. Time lapse series showing the nucleation, growth and removal of a secondary lipid bilayer. The formation of the second layer is highlighted by the yellow arrows. Scale bar: 10 µm. The intensity step height reveals a proportional increase in fluorescence intensity, namely, 1 layer ~ 2000 a.u. and 2 layers ~ 4000 a.u.

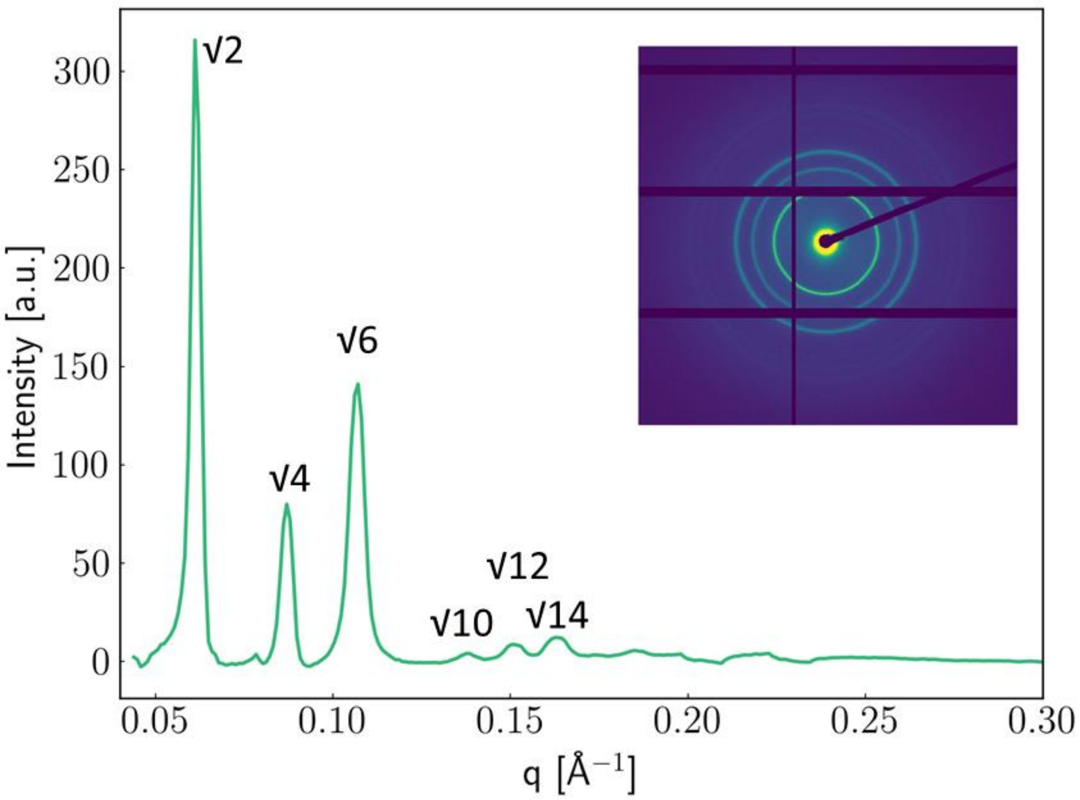

Supplementary Figure 4. SAXS 1D diffraction pattern for MO cubosomes. The inset includes the corresponding 2D image.

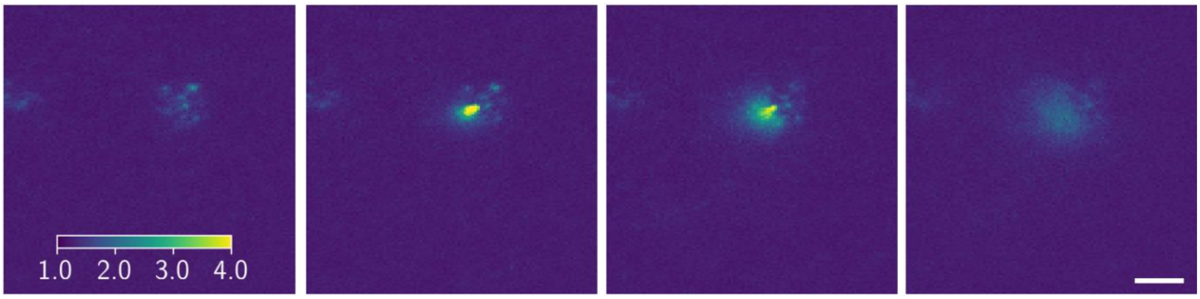

Supplementary Figure 5. Asymmetric mixing behaviour observed for a cubosome landing on the edge between two distinct domains. Scale bar: 2 µm.

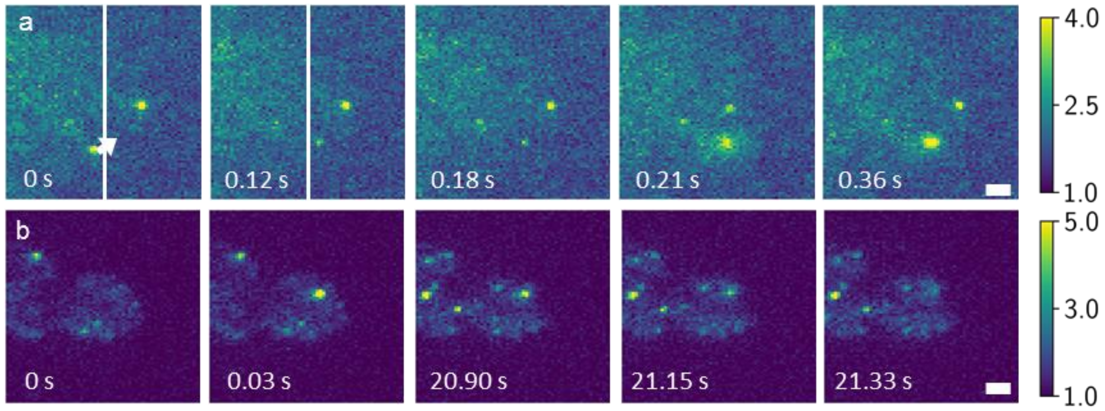

Supplementary Figure 6. Time lapse series fusion events between a cubosome and SLB. (a) Highlights a cubosome immobilizing at the surface before 'jumping' and rapidly fusing at an adjacent location. The vertical white line is a reference, emphasizing the movement from the left to the right of the line. (b) Highlights a cubosome fusing with a DOPS rich domain. This event demonstrates slow then fast fusion. The far right cubosome lands, persists for 20 s, before suddenly fusing with the bilayer. Scale bar = 1 µm.

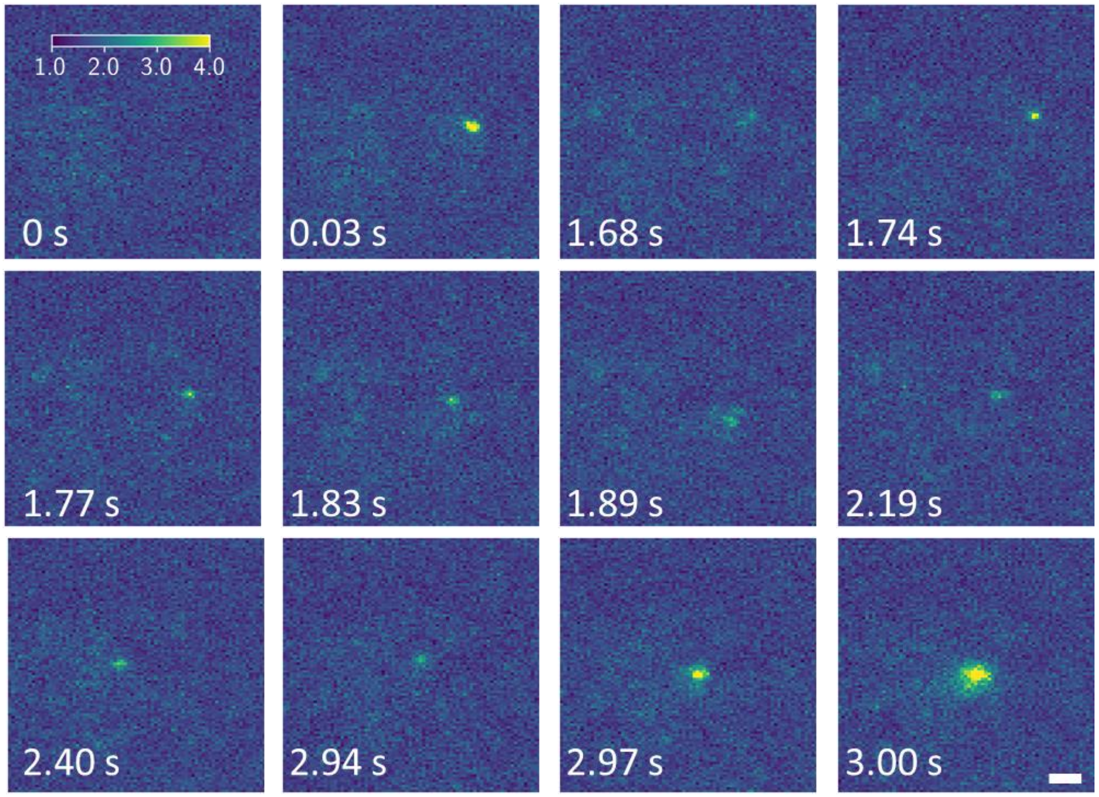

Supplementary Figure 7. Time lapse series showing the individual cubosome attachment and release from a DOPC bilayer. In this case the cubosome is immobilized on the SLB at 0.03 s before releasing at 1.68 s. The cubosome diffuses above the surface before landing at another location and undergoing fusion with the SLB. Scale bar = 1 µm.

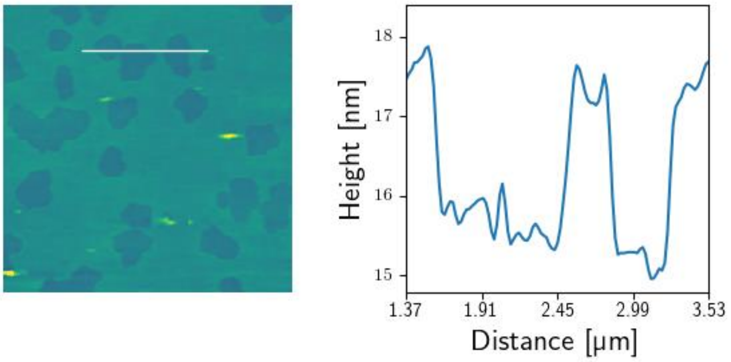

Supplementary Figure 8. AFM height image and corresponding line profile of a mixed DOPS/DOPC bilayer. The variation in step height is shown to be ~ 2 nm.

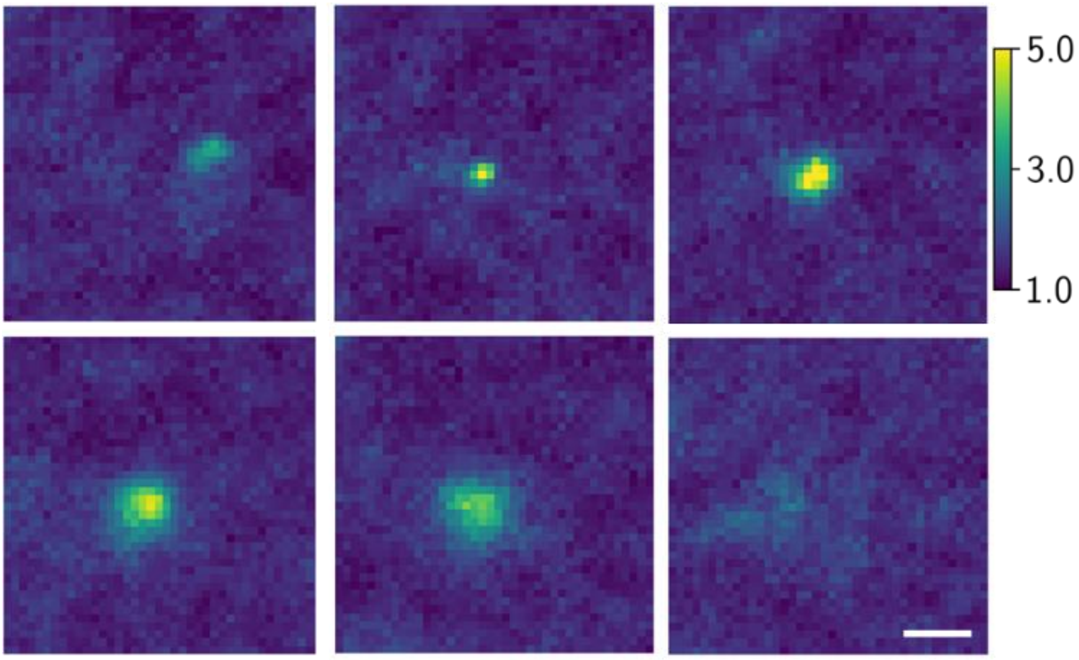

Supplementary Figure 9. Fusion of MO cubosomes stained by phospholipid dye (18:1 Liss Rhod PE) with DOPC bilayer. Scale bar = 1.5 µm.

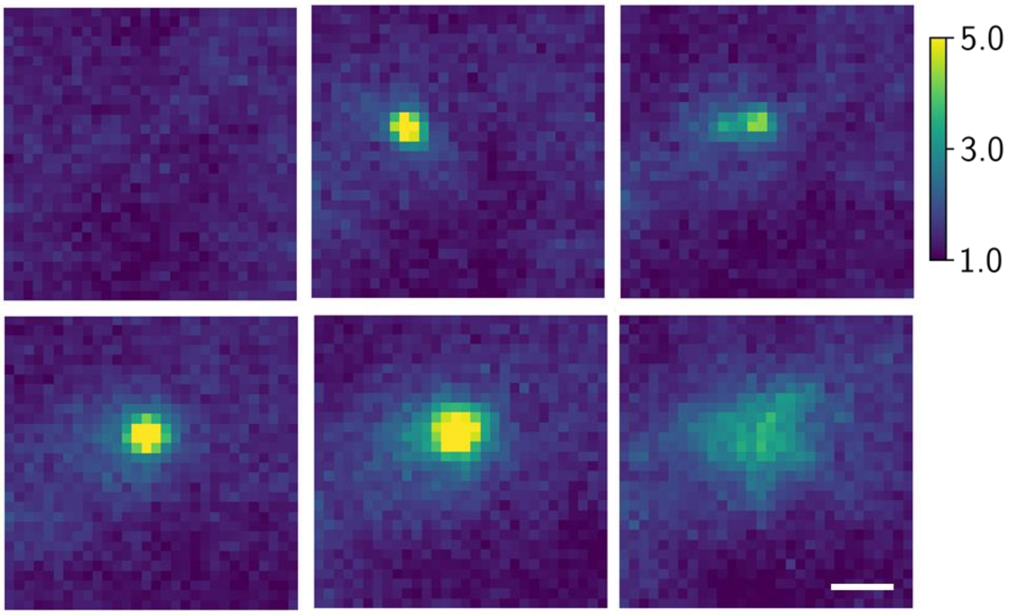

Supplementary Figure 10. Fusion of MO cubosomes stained by phospholipid dye (18:1 Liss Rhod PE) with DOPC bilayer. Scale bar = 1.5 μm.

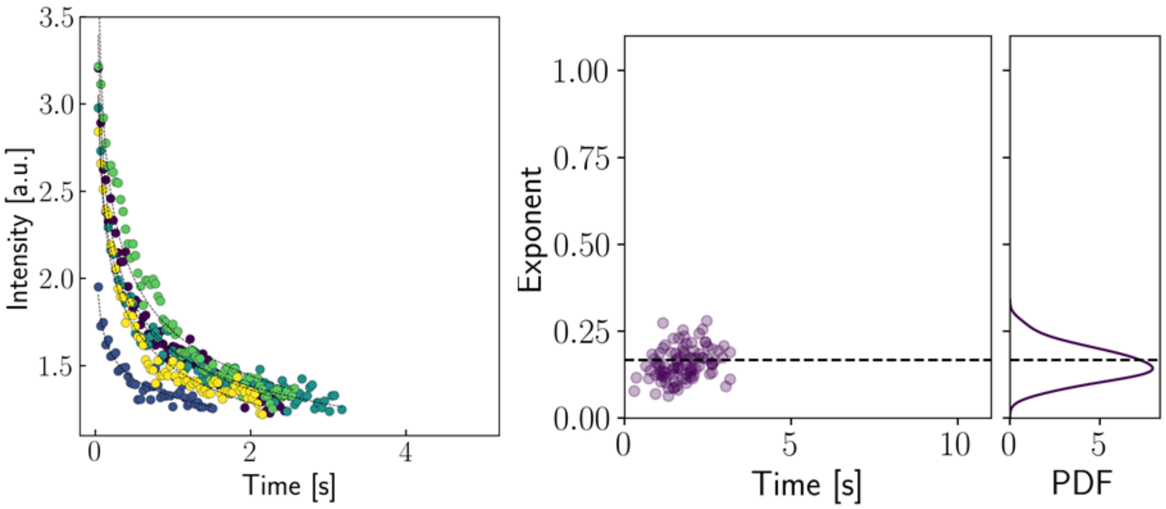

Supplementary Figure 11. Representative intensity plots (five shown) and scaling for MO cubosomes stained by phospholipid dye (18:1 Liss Rhod PE) fusing with DOPC bilayer.

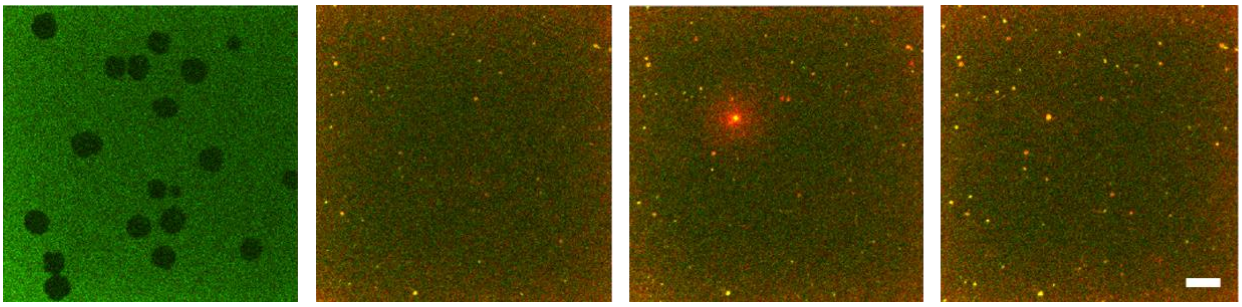

Supplementary Figure 12. Confocal microscopy images highlighting the mutual diffusion in late stages of cubosome introduction to SLBs. The SLB is initiated dyed green and the cubosome is loaded with red dye. The latter three frames show at the later stages of the experiment that the cubosomes persist on the surface. Red dye is delivered into the bilayer and green dye extracted into the cubosomes. Scale bar: 10 µm.

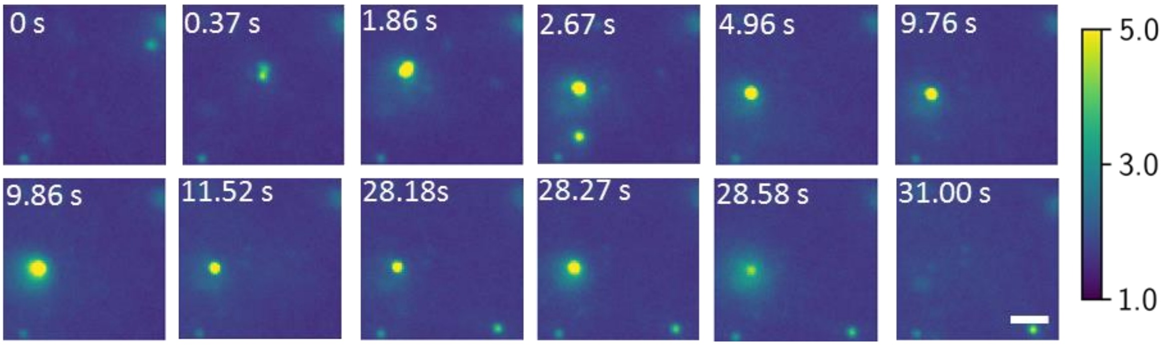

Supplementary Figure 13. TIRF snapshots of a cubosome fusion event between 1% DOTAP and 40% DOPS 30% CHOL within BSA-PBS environment. In this case a small fusion event was observed at frames 9.76-9.86s before a typical fusion event was again observed at ~ 28 s.
